# The Beat

**DOI:** 10.1289/ehp.121-a119

**Published:** 2013-04-01

**Authors:** Erin E. Dooley

**Affiliations:** Erin E. Dooley, MA, is a staff writer for *EHP*.

## Update for Science Education Standards

New national standards for K–12 science education have been finalized and are set for release in spring 2013.^1^ The Next Generation Science Standards were developed by the National Research Council, the National Science Teachers Association, the American Association for the Advancement of Science, the nonprofit Achieve, and 26 states. The standards focus less on rote memorization and more on the practice of science, with an emphasis on college readiness. “There hasn’t been a national effort to update science standards in over fifteen years,” says Chad Colby, communications director for Achieve. “But the science has changed, and the science about how students learn has changed.” Some textbook publishers may incorporate the new standards into their materials as early as next year.

**Figure:**
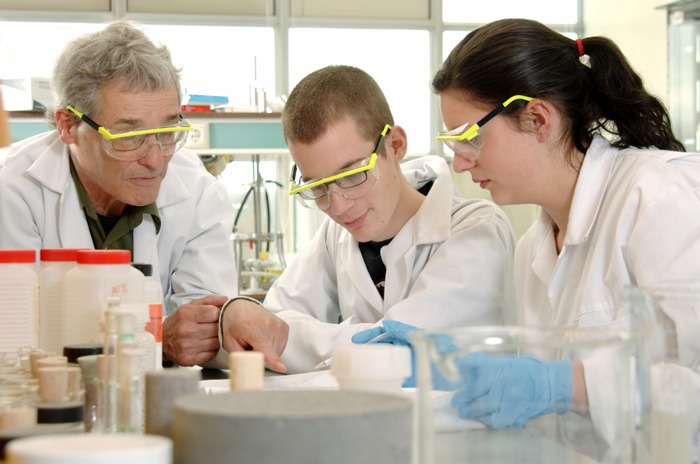
Coming soon to a classroom near you: new national science education standards. iStockphoto.com

## North Carolina Considering Underground Disposal of  Fracking Waste

In February 2013 the North Carolina Senate approved a bill that would lift the state’s moratorium on hydraulic fracturing (“fracking”) for oil and gas by 2015 and overturn a 40-year-old ban on underground injection of waste.^2^ The bill, now making its way through the House, would set up an emergency fund to remediate oil spills using state royalties from offshore energy development. The geology of central North Carolina, where fracking would take place, is not amenable to underground injection, so the waste would likely be transported to the eastern area of the state.^3^

## Smoking Bans and Preterm  Birth Rates

A Belgian investigation of birth outcomes before, during, and after smoking bans found an association between implemention of such bans and lower rates of preterm birth.^4^ The reductions followed a pattern consistent with the three-phase introduction of smoking bans in public places/workplaces, in restaurants, and in bars serving food, respectively. No such trend was seen in the years before the bans. Preterm birth, defined as a birth occurring before 37 weeks of pregnancy, has been linked with adverse health effects across a child’s life span.

**Figure:**
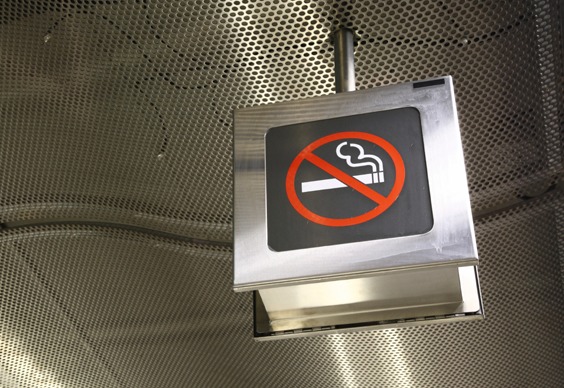
Gradually stricter smoking bans in Belgium tracked with gradual reductions in preterm birth weights. iStockphoto.com

## Take Care When Gardening Near Contaminated Sites

A study conducted near an Arizona Superfund site found that certain families of vegetables are especially effective at accumulating arsenic from soil, with the Asteraceae (e.g., lettuces) and Brassicaceae (e.g., radishes, broccoli, cabbage, and kale) families of vegetables packing the most arsenic into their edible portions.^5^ The study authors recommend that gardeners in areas with naturally high levels of arsenic or those near smelter or mining sites test their soil prior to gardening. They also advise gardeners who live in high-arsenic areas to limit their intake of homegrown vegetables, especially vegetables in the two hyperaccumulator families.

**Figure:**
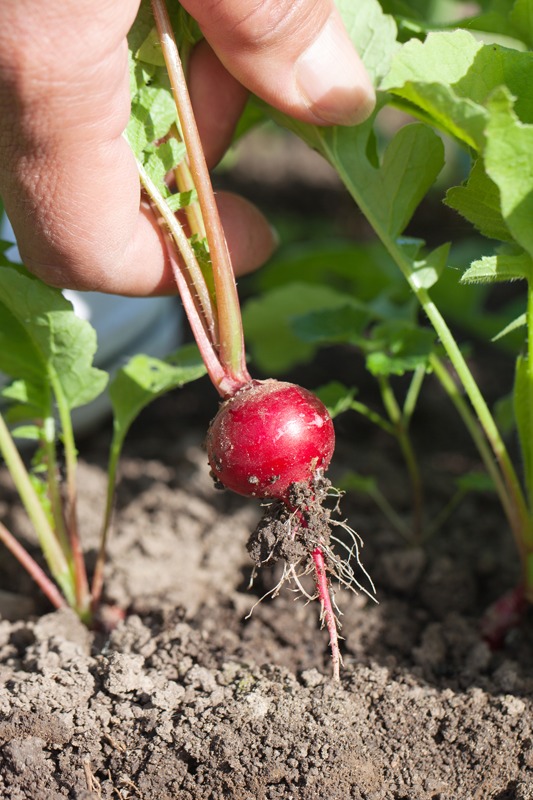
Radishes and other members of the Brassicaceae family have been shown to accumulate high levels of arsenic. © Shutterstock.com / Janis Smits

## Minnesota State Government Phases Out Triclosan Soaps

In March 2013 the Minnesota Pollution Control Agency announced that within three months state agencies will no longer purchase hand, dish, or laundry soaps containing triclosan.^6^ This antibacterial agent is a suspected endocrine disruptor and is believed to contribute to antibiotic resistance.^7^ It is used in a variety of consumer products including soaps, personal care products, cleaning products, fabrics, and plastics. The U.S. FDA, which is conducting an ongoing scientific and regulatory review of triclosan, states that although the compound does provide health benefits in some products (such as toothpaste), there is no evidence that it is any more effective than plain soap and water for bathing or handwashing.^8^
